# Copy Number Variation of the Beta Defensin Gene Cluster on Chromosome 8p Influences the Bacterial Microbiota within the Nasopharynx of Otitis-Prone Children

**DOI:** 10.1371/journal.pone.0098269

**Published:** 2014-05-27

**Authors:** Eric A. Jones, Anchasa Kananurak, Charles L. Bevins, Edward J. Hollox, Lauren O. Bakaletz

**Affiliations:** 1 Center for Microbial Pathogenesis, Department of Pediatrics, College of Medicine, The Research Institute at Nationwide Children's Hospital, The Ohio State University, Columbus, Ohio, United States of America; 2 Department of Microbiology and Immunology, School of Medicine, University of California Davis, Davis, California, United States of America; 3 Department of Genetics, University of Leicester, Leicester, United Kingdom; University Hospital Schleswig-Holstein, Campus Kiel, Germany

## Abstract

As there is increasing evidence that aberrant defensin expression is related to susceptibility for infectious disease and inflammatory disorders, we sought to determine if copy number of the beta-defensin gene cluster located on chromosome 8p23.1 (*DEFB107*, *106*, *105*, *104*, *103*, *DEFB4* and *SPAG11*), that shows copy number variation as a block, was associated with susceptibility to otitis media (OM). The gene *DEFB103* within this complex encodes human beta defensin-3 (hBD-3), an antimicrobial peptide (AP) expressed by epithelial cells that line the mammalian airway, important for defense of mucosal surfaces and previously shown to have bactericidal activity *in vitro* against multiple human pathogens, including the three that predominate in OM. To this end, we conducted a retrospective case-control study of 113 OM prone children and 267 controls aged five to sixty months. We identified the copy number of the above defined beta-defensin gene cluster (DEFB-CN) in each study subject by paralogue ratio assays. The mean DEFB-CN was indistinguishable between subjects classified as OM prone based on a recent history of multiple episodes of OM and control subjects who had no history of OM (4.4±0.96 *versus* 4.4±1.08, respectively: Odds Ratio [OR]: 1.16 (95% CI: 0.61, 2.20). Despite a lack of direct association, we observed a statistically significant correlation between DEFB-CN and nasopharyngeal bacterial colonization patterns. Collectively, our findings suggested that susceptibility to OM might be mediated by genetic variation among individuals, wherein a DEFB-CN less than 4 exerts a marked influence on the microbiota of the nasopharynx, specifically with regard to colonization by the three predominant bacterial pathogens of OM.

## Introduction

Otitis media (OM) is one of the most common diseases of childhood [1]. Accordingly, OM represents the leading indication for antibiotic prescription [2]–[4], pediatric surgery under general anesthesia [5] and deafness in childhood [6]. Costs associated with management of this disease exceed 5 billion dollars annually in the United States alone [2], [7]. At least 80% of children will have experienced one or more episodes of OM by age 3 and more than 50% will have ≥3 episodes [8]. Whereas these statistics show that OM is common across the population, approximately 10–15% of children are considered ‘otitis prone’ due to their even greater incidence of disease [9]. Children that experience ≥3 episodes within the past six months or ≥4 episodes within the past 12 months, with at least 1 episode in the past 6 months are classified as ‘OM prone’ [10].

There are multiple risk factors associated with OM, including environmental factors, antecedent viral infection, male gender and inheritance [11], [12]. Indeed, studies on mono- and dizygotic twins and triplets show a heritability of 57% for acute OM and 72% for chronic OM, emphasizing the importance of genetic variation in contributing to the overall susceptibility to OM [13], [14]. Impaired immunity predisposes children to OM. For example, variation in the immune response genes *TNFA*, *IL6*, *IL10*, *TLR4*, genes encoding various surfactant proteins, mannose binding lectin and immunoglobulins, among others, have been associated with susceptibility to OM [9], [14]–[16]. These associations are not unexpected given that the middle ear is protected from bacterial invasion by multiple mechanisms, including effectors of both innate and acquired immunity.

One category of innate immune effectors operational in the middle ear and Eustachian tube are the antimicrobial host defense peptides, which include the defensin family [17]–[19]. Defensins are cationic peptides with immunomodulatory properties and potent anti-microbial activity against bacteria, viruses, and fungi [17], [20]. One class, the beta-defensins, is secreted by mucosal epithelial cells that line the airway, including the middle ear [18], [21]. Moreover, human beta-defensin-3 (hBD-3) is expressed in the middle ear during OM and is bactericidal for all three predominant causative agents of OM [e.g. nontypeable *Haemophilus influenzae* (NTHI), *Streptococcus pneumoniae* and *Moraxella catarrhalis*], both *in vitro* [22] and *in vivo* [23]. While expression of hBD-3 is important for defense against infectious agents, this antimicrobial peptide likely also plays a key role in maintenance of microbial homeostasis, as virus-mediated dysregulation of hBD-3 expression results in augmented colonization of the uppermost airway by NTHI [23]. This increase in bacterial load in the nasopharynx (NP), coincident with an upper respiratory tract (URT) viral infection, is another known risk factor for OM [24].

The gene encoding hBD-3 (*DEFB103*) resides on chromosome 8p23.1 within in a cluster of seven beta-defensin genes that includes *DEFB107, 106, 105, 104, 103, DEFB4* and *SPAG11*. This cluster forms a large repeat unit, which is variable in copy number as a block (DEFB-CN). The copy number variation for this gene cluster among individuals is usually between 2 and 6, however copy numbers up to 12 have been documented [25]. Copy number variation of beta-defensin genes has been linked with concordant functional expression of specific beta-defensins [26] and associated with important biological consequences relevant to disease [17]. An increased DEFB-CN has been associated with psoriasis [25], [27] and chronic obstructive pulmonary disease [28]. Conversely, a reduced DEFB-CN is associated with susceptibility to celiac disease [29], chronic pancreatitis [30] and necrotizing enterocolitis [31], possibly as a result of an expected overall deficit in beta-defensin production. There are conflicting reports on the association of DEFB-CN with Crohn's disease [32], with data supporting either lower [33] or higher [34] DEFB-CN.

The goal of our study was to investigate possible associations between DEFB-CN and proneness to OM. As such, we sought to characterize bacterial colonization patterns of the pediatric NP and determine whether relative DEFB-CN predisposes to differential composition of the NP microbiota in OM prone children, relative to the three predominant bacterial pathogens of this highly prevalent pediatric disease.

## Materials and Methods

### Ethics Statement

The Institutional Review Board (IRB) at Nationwide Children's Hospital approved the study protocol. Informed, written consent was obtained from the legal guardian of all children prior to sample collection. Due to the minimally invasive nature of NP flora collection, participating control subjects were nominally compensated.

### Experimental Design

To participate, children needed to be between 6 months and 5 years of age at the time of recruitment. Control subjects had no history of recurrent ear infection, no current middle ear or respiratory tract infection and were not on antibiotic therapy; those who did not meet these criteria were excluded. Episodic OM was classified upon evaluation by a physician at Nationwide Children's Hospital; Columbus, Ohio. From this group, ‘OM-prone’ patients were defined as those children who experienced ≥ three episodes of acute OM within the past six months or ≥ four episodes of acute OM within the previous 12 months, with at least one of these episodes occurring in the previous 6 months [10]. OM-prone children undergoing tympanostomy tube insertion served as cases for this study. During tube insertion, a blood or saliva sample was also collected. Blood samples were collected by venipuncture while the patient was anesthetized, then stored at −80°C until genomic DNA (gDNA) was extracted.

To characterize NP bacterial flora and detect the presence of specific bacterial pathogens within the middle ear of cases, NP swabs (CultureSwab plus Amies Gel without Charcoal, BD Diagnostic Systems) from both nares, and middle ear fluids, when present, were collected during tympanostomy tube insertion. For middle ear fluid collection, Juhn Tymp taps (Xomed Treace Products, Jacksonville, FL; USA) were used. Subject samples were stored on ice for transport to the clinical microbiology laboratory for microbe identification.

Control subjects were recruited during either well-child visits to Close-to-Home Centers of Nationwide Children's Hospital system or during random visits to the Center for Science and Industry (COSI); Columbus, Ohio. At enrollment a blood or saliva sample was collected for recovery of gDNA and NP swabs were taken via both nares.

### Bacteriological Data

To detect the presence of *S. pneumoniae, H. influenzae*, or *M. catarrhalis, c*ulture data was obtained following selective plating of NP swabs and middle ear fluids with incubation overnight at 37°C, in a humidified atmosphere of 5% CO_2_. *Haemophilus* X-V factor test (Fisher Scientific, CA; USA) was utilized and an isolate was considered to be *H. influenzae* if growth was observed between the two strips. *H. influenzae* isolates were further characterized as being nontypeable if non-reactive with specific antibody directed against capsule types a-f. To identify an isolate as *M. catarrhalis*, isolates were subjected to an Mcat disk test (Fisher Scientific, CA; USA), according to manufacturer's protocol. *S. pneumoniae* isolates were identified via Ox Bial test (Fisher Scientific, CA; USA) according to supplied instructions. Patients were identified as culture positive if plating of NP swabs resulted in bacterial growth on one or more selective media. Once a positive identification was made, isolated colonies were frozen in a skim milk/15% glycerol solution and stored at −80°C.

### DNA Isolation

Genomic DNA was extracted from either blood or saliva, then sent as coded samples to U.C. Davis for evaluation. Saliva samples were collected using Oragene DNA saliva collection kit (DNA Genotek, Ontario, Canada) according to the manufacturer's protocol. Genomic DNA was extracted from peripheral blood leukocytes using the Qiagen QIAamp DNA Blood Reagent Kit according to the manufacturer's protocol. For PCR analysis, genomic DNA concentrations were quantified by spectrophotometry at 260 nm using a Nanodrop spectrometer then diluted to 10 ng/µl in 50.0 µM Tris buffer (pH 8.0) containing yeast RNA (20 µg/ml, functioning as a nucleic acid carrier).

### Copy Number Determination

Using previously published methods [32], [35], [36], DEFB-CN was determined by co-authors (AK and CB) who were blinded as to case-control status and colonization data. Briefly, the measurement of DEFB-CN utilized three paralogue ratio test (PRT) assays: i) PRT107A, simultaneously amplifies a locus adjacent to *DEFB107* and an unlinked reference locus on chromosome 11, ii) HSPD21, amplifies a pseudogene located within the beta-defensin repeat unit, and an unlinked reference locus on chromosome 21, and iii) 5del, amplifies a multi-allelic insertion/deletion (indel) segments (rs5889219) located within the beta-defensin repeat unit [37]. The ratio of test to reference provided an accurate estimate of the copy number. The 5del assay is a multi-allelic ratio test that provided the closest integer number for gene copy number when the other two assays were ambiguous. Positive control DNAs were six samples (C0207, C0088, C0849, C0940, C0969, and C0913) from the Human Random Control DNA Panels from European Collection of Cell Cultures (ECACC) commercially available through Sigma Chemicals [HRC-1 (2 µ7 g)], with expected DEFB-CNs of 5, 4, 6, 4, 5, and 3, respectively. The 96-well assay plates contained randomly interspersed PRT reactions for reference DNA samples, experimental samples and no template controls.

Using ROX500 standard marker, raw data from the fragment analysis was analyzed with PeakScanner Software v1.0 (Applied Biosystems). Data was filtered for product sizes of: 153 and 155 for PRT107A; 170 and 178 for HSPD21; and 3 potential product sizes of 123, 125, 127 for 5del. To demonstrate concordance in duplicate independent assays, each PCR was performed using one of two different fluorescently labeled primers (NED- or FAM-labeled for PRT107, HEX- or FAM-labeled for HSPD, HEX- or FAM-labeled for 5del). Aliquots of all six reaction products were combined for capillary electrophoresis analysis. The copy number estimates from the PRT107A and HSPD21 assays were combined using a maximum-likelihood method, which incorporated a regression analysis using the gene copy number values from the reference control DNA assays. The resulting linear regression calibrated the gene copy number estimates for the experimental samples, together with an associated significance value reflecting the confidence for that estimate of gene copy number compared to any other copy numbers estimate. In cases where the combined PRT107A and HSPD21 assays returned a non-integer copy number, the 5del assay data provided two additional gene copy number estimates that were weighted according to the variability of all six individual assays [36], [37].

### Statistical Analysis

Associations between DEFB-CN, case-control status, and bacterial colonization of the NP, were determined by Fisher's exact test and univariate logistic regression with correction for multiple testing via Tukey's method for multiple comparisons. Mean DEFB-CN among cases and controls were compared using an unpaired, two-tailed t-test. For the purposes of analysis, DEFB-CN was treated as a categorical variable, where subjects were placed into one of two distinct groups; subjects having DEFB-CN ≤4 or separately, subjects with an observed DEFB-CN >4. Age was also treated as a categorical variable, with subjects divided into two groups, those age ≤24 months and all subjects >24 months of age comprising a second, distinct group. In all cases, a *p*-value <0.05 was considered statistically significant. All statistical analyses were performed with GraphPad Prism version 5.0 (GraphPad Software, La Jolla, CA, USA).

## Results

### Subject characteristics

A total of 372 children met inclusion criteria (Table 1). Participants ranged from 6-50 months of age, with a mean of 25.9±12.8 months. A total of 113 children were classified as OM prone whereas 259 subjects served as controls. The mean age among OM prone subjects was 21.3±11.7 months and 27.9±12.7 months for controls.

**Table 1:** Subject Demographics.

Variable	OM Prone	Healthy Control	Total Cohort
Subjects	N = 113	N = 259	N = 372
Mean DEFB-CN±S.D. (Range)	4±0.96 (2–9)	4±1.08 (2–9)	4±1.04 (2–9)
Mean Age in Months ±S.D. (Range)	21.3±11.7 (6–50)	27.9±12.7 (6–50)	25.9±12.8 (6–50)
Male n (%)	73 (66)	146 (56)	219 (59)
Ethnicity n (%)			
African American	14 (12)	10 (4)	24 (6.9)
Asian	1 (0.1)	15 (6)	16 (4)
Caucasian	97 (86)	231 (89)	328 (89)
Hispanic	0 (0)	2 (0.07)	2 (0.05)
Unknown	1 (0.1)	1 (0.03)	2 (0.05)

### Observed DEFB-CN between OM prone and control subjects

**Figure 1:**
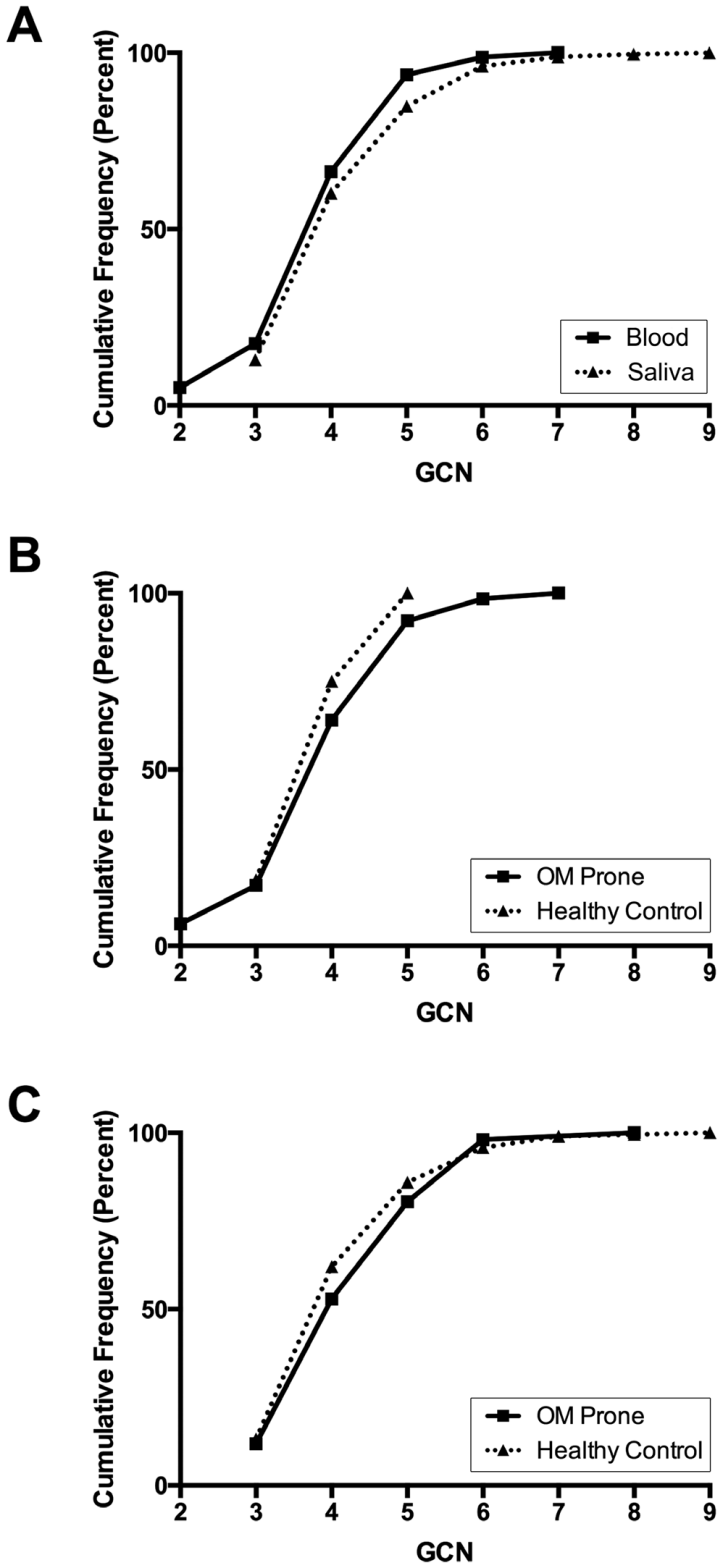
Cumulative frequency (percent) distribution of raw DEFB-CN determined from DNA obtained from blood and saliva samples. (Panel A) We observed a slightly lower mean DEFB-CN among gDNA templates isolated from whole blood samples (—_█_—) relative to DEFB-CN estimated from gDNA extracted from saliva samples (^•••^▴^•••^) (*p*<0.05, t-test). However, when stratified by sample type, there were no significant differences found between OM prone (—_█_—) and control (^•••^▴^•••^) DEFB-CN derived from either whole blood samples (Panel B) (*p* = 0.55, t-test) or saliva samples (Panel C) (*p* = 0.37, t-test).

To quantify DEFB-CN, gDNA extracted from blood or saliva samples we used three PRT assays, as described in Methods. Of the 372 enrolled participants, we obtained reliable DEFB-CN values for 343 (92%). Of these 343 samples, 80 (23%) were whole blood and 263 (77%) were saliva. We observed a slight difference in DEFB-CN values derived from gDNA depending on the biological source of the sample, with overall slightly lower DEFB-CN detected from whole blood (*p*<0.05, t-test) (**Fig. 1A**). We suspect that this result is driven by a sampling effect: the absence of rarer high copy number samples in the blood cohort due to the smaller sample size of this cohort as has been observed in other studies [38]. However, when stratified by sample type, there were no significant differences found between OM prone and control DEFB-CN derived from whole blood (*p* = 0.55, t-test) or saliva samples (*p* = 0.37, t-test) (**Figs. 1B&C**).

**Figure 2:**
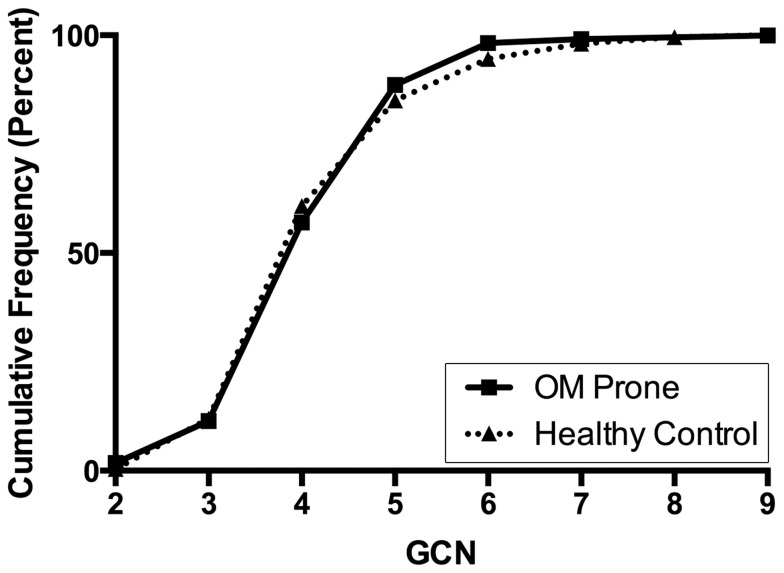
Cumulative DEFB-CN frequency (percent) distribution among OM prone and control subjects. The overall distribution of DEFB-CN did not differ significantly among OM prone (—_█_—) or control (**^•••^**▴**^•••^**) study subjects (p>0.05).

Among study subjects, DEFB-CN ranged from 2 to 9 copies per diploid genome (Table 1). The mean DEFB-CN for the entire cohort was 4±1.04; for OM prone children 4±0.96; and for controls 4±1.08. The modal DEFB-CN for both groups was 4 copies, as expected [25] (**Fig. 2**). We observed no significant difference between mean DEFB-CN between the ‘OM prone’ and control groups (*p*>0.05) (**Table 1**).

### Characterization of nasopharyngeal bacterial colonization patterns between OM prone and control subjects

NP swabs were used to isolate and identify specific patterns of bacterial colonization of the NP due to the three predominant pathogens of OM. Of the 372 participants, 30 (8%) were colonized with NTHI, *M. catarrhalis* (Mcat) and *S. pneumoniae*, concurrently (**Table 2**). NTHI was detected in the NP of 101 (27%), *S. pneumoniae* in 201 (54%) and Mcat in 143 (41%). Additionally, dual colonization by NTHI and Mcat was detected in 39 (10%) subjects; NTHI and *S. pneumoniae* in 73 subjects (19%) and *S. pneumoniae* plus Mcat in 95 subjects (26%). In addition to colonization by multiple pathogens, among OM prone subjects, 54 (55%) were culture positive for NTHI alone, 51 (36%) were culture positive for Mcat alone and 77 (38%) were culture positive for *S. pneumoniae* alone.

**Table 2:** Nasopharyngeal colonization pattern among study subjects.

	*Spn	*NTHI	Mcat	*Spn/NTHI/Mcat	Spn/NTHI	Spn/Mcat	*NTHI/Mcat
OM Prone n(%)	77 (38)	54 (55)	51 (36)	20 (68)	40 (55)	36 (38)	26 (67)
Healthy Control n(%)	124 (62)	47 (45)	92 (64)	10 (32)	33 (45)	59 (62)	13 (33)
Total	201	101	143	30	73	95	39

*Indicates a significant difference (*p*-value <0.05) between OM prone and healthy controls.

When bacterial colonization patterns were restricted by case-control status, concurrent colonization by NTHI, *M. catarrhalis* and *S. pneumoniae* differed significantly between OM prone and control children (68% vs. 32%) *p*<0.001. Dual colonization by NTHI and Mcat also differed significantly between OM prone and controls, wherein 26 (67%) OM prone children were culture positive for these two organisms, while only 13 (33%) controls exhibited this same colonization pattern (*p*<0.001). Likewise, 53 (55%) OM prone children were culture positive for NTHI *versus* 43 (45%) among controls. This finding was also statistically significant (*p*<0.001) (**Table 2**). Interestingly, for colonization by Spn alone, this patterns was reversed with 124 (62%) of healthy children being culture positive *versus* 77 (38%) of OM prone children (*p* = 0.0002).

### DEFB-CN and OM proneness are predictors of NP colonization patterns

To identify variables associated with DEFB-CN and disease among subjects, we conducted logistic regression analyses of various subject-level variables within our study cohort. We found no evidence for a direct statistical association between DEFB-CN and disease status (*p*>0.05). Thusly, as the modal DEFB-CN within our cohort was determined to be 4, we stratified subjects based upon those with a DEFB-CN≥4 *versus* all other subjects, in order to identify further associations of interest. When stratified in this manner, we observed that neither single colonization with NTHI (*p* = 0.59), Mcat (*p* = 0.52) or *S. pneumoniae* (*p* = 0.24) was associated with DEFB-CN status. However, subjects with a DEFB-CNless than 4 were significantly more likely to be colonized by NTHI, *S. pneumoniae* and Mcat concurrently (*p* = 0.03). In addition, having a DEFB-CN less than 4 significantly increased the likelihood of dual colonization by NTHI and Mcat (*p* = 0.05), as well as by *S. pneumoniae* and Mcat (*p* = 0.03) (**Table 3**).

**Table 3:** Univariate analyses of subject-level characteristics.

	Odds ratio (95% CI)	*p* -Value
DEFB-CN≤4		
Disease Status	1.16 (0.61, 2.20)	0.74
NTHI/Mcat/Spn	2.83 (1.16, 6.86)	0.03
NTHI	1.24 (0.64, 2.41)	0.59
Spn	1.52 (0.77, 3.01)	0.24
Mcat	1.27 (0.68, 2.36)	0.52
NTHI/Mcat	2.88 (1.09, 7.63)	0.05
Spn/Mcat	2.11 (1.11, 4.00)	0.03
Disease Status		
NTHI/Mcat/Spn	5.33 (2.41, 11.87)	<0.0001
NTHI	4.12 (2.53, 6.71)	<0.0001
Spn	1.33 (0.83, 2.13)	0.24
Mcat	1.31 (0.83, 2.05)	0.25
NTHI/Mcat	5.65 (2.78, 11.50)	<0.0001
NTHI/Spn	3.88 (2.26, 6.65)	<0.0001
Male Gender	1.41 (0.89, 2.23)	0.17
Age ≤24 mos.	2.89 (1.73, 4.85)	<0.0001

To determine if bacterial colonization of the NP was significantly associated with OM proneness, we additionally stratified upon this variable and subsequently tested for statistical significance of NP colonization patterns between OM prone and control subjects. Concurrent colonization by NTHI, *S. pneumoniae* and Mcat was a statistically significant predictor of OM prone case status (*p*<0.0001), as was colonization by NTHI alone (*p*<0.0001). Further, dual colonization by NTHI and Mcat (*p*<0.0001) as well as dual colonization by NTHI and *S. pneumoniae* (*p*<0.0001); were statistically significant predictors of being classified as OM prone. Neither colonization of the NP by *S. pneumoniae* (*p* = 0.24) or Mcat (*p* = 0.25) alone were associated with OM prone case status in our study (**Table 3**). Whereas being male was not associated with OM prone case status (*p* = 0.17), being ≤24 months of age was associated with such status (*p*<0.0001).

## Discussion

Frequent bouts of OM are associated with an overall diminished quality of life [39]. Owing to the multifactorial and polymicrobial nature of acute, chronic and recurrent OM, this spectrum of diseases will never be attributed to a single risk factor. However, gaining a better understanding of the specific risk factors which predispose a child to recurrent episodes of OM would significantly improve our ability to diagnose and design medical interventions to proactively manage this prevalent pediatric disease and thereby lessen the enormous associated socioeconomic burden. Due to our interest in the polymicrobial nature of OM, as well as the fact that colonization of the NP is the first step in the disease process [40], we decided to further investigate risk factors which may help shape NP colonization patterns in children, particularly those deemed OM-prone.

Children are colonized by various bacteria early in life [41], [42]. Among those known to colonize the pediatric NP early, *S. pneumoniae*, *M. catarrhalis* and NTHI are of critical import to our understanding of OM pathogenesis. By age 1, up to 54% of children are colonized with *S. pneumoniae*, 74% with *M. catarrhalis* and 33% with NTHI [43]. However, it is also known that the presence or absence of *specific* members of the NP microbiota has a direct impact on the incidence of OM [41], [44], [45] and further, that host factors, including relative expression of innate immune effectors such as defensins, may shape the microbial ecology of commensal organisms [17]. Pettigrew and colleagues [46] demonstrated an increased incidence of *H*. *influenzae* colonization among children with episodic OM as well as a negative association between *S. pneumoniae* and *H. influenzae* colonization, which has also been observed by Casey et al. [47]. Moreover, a negative relationship between *S. pneumoniae* colonization and bacterial diversity within the NP during OM has been shown and likewise, specific bacterial colonization patterns have been associated with risk of OM [48] Thus, the specific makeup of commensal organisms within the NP micro-environment is likely playing an important role in establishing differential susceptibility to infectious diseases of the airway.

As bacterial invasion of the middle ear from the NP is often triggered by a previous or ongoing viral URT infection [49], it is also important to consider the effects that viral co-infection have on NP colonization. We showed, in an animal model of OM, that one mechanism which facilitates invasion of the middle ear by NTHI is viral dysregulation of host defensin expression. Further, neutralization of beta-defensin led to increased NTHI bacterial load within the chinchilla NP [23]. Collectively, considering that: 1) the composition of bacteria resident within the pediatric NP is associated with risk of OM; 2) URT viral infection is coincident with development of bacterial OM; 3) viral predisposition to bacterial invasion of the middle ear can be mediated by dysregulation of expression of innate immune effectors; and 4) dysregulated expression of even a single AP can lead to an increased bacterial load in the NP, we sought to investigate whether genetic polymorphisms in beta-defensins contributed to susceptibility to OM. Here, we tested if polymorphism in the copy number of the beta defensin gene cluster on chromosome 8pwas associated with proneness to OM, perhaps via influence on composition of bacteria colonizing the pediatric NP.

Overall, when comparing control subjects to a group defined as ‘OM prone’, we observed no direct association with DEFB-CN. However, DEFB-CN was associated with composition of the NP microbiota with regard to the three predominant pathogens of OM. Thus, our data revealed an underlying genetic mechanism for OM proneness via imparted NP colonization patterns. Sixty-eight percent of all OM prone children were culture positive for concurrent colonization by the three predominant pathogens of OM, whereas only 32% percent of controls were so colonized, suggesting an important role for this specific NP colonization pattern with regards to OM proneness. Our data suggested that a DEFB-CN less than 4 increased the risk for concurrent NTHI, *S. pneumoniae* and *M. catarrhalis* colonization, as well as dual colonization by either NTHI and *M. catarrhalis* or NTHI and *S. pneumoniae*. Such colonization patterns were directly linked to OM proneness. These data are in line with earlier reports wherein simultaneous colonization by multiple pathogens of acute OM was associated with a greater risk of disease than was colonization by a single pathogen [50], [51]. With regard to our data, whereas *DEFB103* is part of the gene cluster we analyzed for relative copy number, and thus hBD-3 is implicated in the biology described here, we cannot exclude important contributions by *DEFB4* or another defensin encoded in the repeat unit.

OM prone children were also four times as likely to be colonized with NTHI, supporting previously published studies which found similar associations during URT infections. Also, we observed a decrease in colonization by *S. pneumoniae* relative to NTHI among OM prone subjects. It is theorized this trend is possibly due to recent antibiotic use within this subset of children [52]. Given that OM prone children in our study were recruited at the time of tympanometry tube insertion, these data fit well with such a hypothesis. In totality, our data not only support published findings demonstrating that the composition of microbiota within the NP has significant bearing on susceptibility to URT infections, including OM in children, but identify a genetic factor that underlies the composition of bacteria within this anatomical niche, specifically with regard to the predominant pathogens of OM. As such, our data further support the notion for dual roles among defensins in human health; namely, antimicrobial activity against pathogenic organisms, as well as active management of the microbial ecology of commensals [17], [53].

To the best of our knowledge, this is the first example of an association between DEFB-CN and three members of the colonizing bacterial microbiota within a specific anatomic niche, here the pediatric nasopharynx. The implication being that prospective determination of a child's DEFB-CN, together with identification of other OM risk factors, could provide an opportunity for a personalized approach to medical management of OM in childhood, perhaps leading to more rational use of antibiotics and reduced need for costly and painful surgical intervention associated with this highly prevalent pediatric infectious disease.
